# The Library's Guide to Sexual and Reproductive Health Information

**DOI:** 10.5195/jmla.2024.1866

**Published:** 2024-07-01

**Authors:** Hilary Michelle Jasmin

**Affiliations:** 1 hjasmin@uthsc.edu, Assistant Professor/Research and Learning Services Librarian, University of Tennessee Health Science Center, Memphis, TN

With the overturning of Roe v. Wade in 2022, Americans had more uncertainty in their reproductive rights than they had in nearly fifty years. With increasing anxiety around health information, it's critical for libraries of all types to prepare adequately for the challenges patrons face when searching for information they may feel shame or embarrassment around. Barbara A. Alvarez's The Library's Guide to Sexual and Reproductive Health Information is a timely, thorough collection of the history of reproductive oppression and injustices and implementation plans for librarians in collections and outreach.

The text is divided into three main sections: Foundation, Education, and Implementation. In Foundation, Alvarez focuses on the fundamentals around sexual and reproductive health (SRH), on SRH as a library service, and on sexuality at large. In Education, Alvarez takes care to separate sexual health and reproductive health into equally essential but different areas of focus, as well as a chapter dedicated to SRH for LGBTQIA+ folks. Finally, Alvarez concludes with implementation plans for libraries, primarily in public library spaces.

Alvarez adeptly takes these enormous concepts and covers them with inclusivity at the front of mind. Reproductive justice is a concept that is highlighted over the course of the text, calling attention to the fact “…that people of color and marginalized communities have long been denied the ability to exercise bodily autonomy and raise their families safely.” (p. 6) Alvarez takes great care to illustrate how SRH is more than abortion or contraception, but “…it is a holistic lens of one's welfare and encompasses topics like housing, community safety, job opportunities, schools, (dis)ability, socioeconomic status, class, race, sexual orientations, and gender identity.” (p. 6) There are very useful blurbs interspersed throughout the content to loop thoughts back toward libraries, covering topics like “Intersectionality in the Stacks,” “Gender Inclusivity at the Public Library,” and “SRH Resource Guide Ideas.”

The audience in mind here is repeatedly public librarians, but there is room for all library types to implement an outreach idea or a resource guide from the Implementation portion of the text. This would fit nicely in a collection that encompasses other health information service textbooks and diverse user out-reach textbooks, like Serving the Underserved (ISBN 978-0-8389-3652-8) or Promoting Individual and Community Health at the Library (ISBN 978-0-8389-1627-8). Even if a reader feels well-versed in SRH, the Implementation portion offers exciting suggestions across all library roles, from collections to outreach to instruction, all while maintaining a lens of information professionals rather than healthcare workers.

In conclusion, The Library's Guide to Sexual and Reproductive Health Information provides a wealth of inclusive information regarding sexual and reproductive health. Though aimed at the public librarian audience, this would be equally valuable to librarians managing collections and outreach efforts in academic libraries, particularly in health sciences.

